# Hemodialysis versus Peritoneal Dialysis: A Comparison of Survival Outcomes in South-East Asian Patients with End-Stage Renal Disease

**DOI:** 10.1371/journal.pone.0140195

**Published:** 2015-10-07

**Authors:** Fan Yang, Lay-Wai Khin, Titus Lau, Horng-Ruey Chua, A. Vathsala, Evan Lee, Nan Luo

**Affiliations:** 1 Saw Swee Hock School of Public Health, National University of Singapore, Singapore, Singapore; 2 Division of Nephrology, University Medicine Cluster, National University Health System, Singapore, Singapore; Medical College of Soochow University, CHINA

## Abstract

**Background:**

Studies comparing patient survival of hemodialysis (HD) and peritoneal dialysis (PD) have yielded conflicting results and no such study was from South-East Asia. This study aimed to compare the survival outcomes of patients with end-stage renal disease (ESRD) who started dialysis with HD and PD in Singapore.

**Methods:**

Survival data for a maximum of 5 years from a single-center cohort of 871 ESRD patients starting dialysis with HD (n = 641) or PD (n = 230) from 2005–2010 was analyzed using the flexible Royston-Parmar (RP) model. The model was also applied to a subsample of 225 propensity-score-matched patient pairs and subgroups defined by age, diabetes mellitus, and cardiovascular disease.

**Results:**

After adjusting for the effect of socio-demographic and clinical characteristics, the risk of death was higher in patients initiating dialysis with PD than those initiating dialysis with HD (hazard ratio [HR]: 2.08; 95% confidence interval [CI]: 1.67–2.59; p<0.001), although there was no significant difference in mortality between the two modalities in the first 12 months of treatment. Consistently, in the matched subsample, patients starting PD had a higher risk of death than those starting HD (HR: 1.73, 95% CI: 1.30–2.28, p<0.001). Subgroup analysis showed that PD may be similar to or better than HD in survival outcomes among young patients (≤65 years old) without diabetes or cardiovascular disease.

**Conclusion:**

ESRD patients who initiated dialysis with HD experienced better survival outcomes than those who initiated dialysis with PD in Singapore, although survival outcomes may not differ between the two dialysis modalities in young and healthier patients. These findings are potentially confounded by selection bias, as patients were not randomized to the two dialysis modalities in this cohort study.

## Introduction

End-stage renal disease (ESRD) has become a significant and growing public health problem worldwide. The global average prevalence of ESRD patients on dialysis was 215 per million population [1], and the total number of dialysis patients in 2010 was estimated to be close to two million [2]. Asians have been reported to have higher prevalence rate of ESRD than Caucasians [3]. In Singapore, the prevalence of ESRD was 1436.1 per million population in 2013 and the number of prevalent dialysis patients increased at an average rate of 8% per year from 1999 to 2013 [4]. The dialysis population is projected to increase sharply due to the nation’s aging population and the high prevalence of diabetes [5].

Hemodialysis (HD) and peritoneal dialysis (PD) are the two common forms of dialysis therapy for ESRD. The mortality of ESRD patients who are treated with the two modalities has been investigated in numerous observational studies [6–13]. But which dialysis modality performs better in prolonging life of ESRD patients is not clear. Some studies showed the superior outcomes of HD [6, 7, 9], whereas others demonstrated that PD was equivalent to HD [12, 13], or even better for certain subgroups [8, 10, 11]. Moreover, the vast majority of these comparisons were done in Western countries; in Asia, similar comparative studies were performed in Taiwan and Korea [11–13]. There was no such study from South-East Asia, home to more than 593 million people.

Therefore, in this study, we aimed to compare the survival outcomes of patients starting different dialysis modalities using a multiethnic ESRD patient cohort obtained from a hospital-based registry in Singapore.

## Methods

### Data

The hospital registry contains data of newly diagnosed ESRD patients in National University Hospital (NUH), Singapore from January 2005 to December 2010. Patients were followed up for a maximum of 5 years (median 3.2 years). Adult patients (≥18-year-old) who began either HD or PD and survived the first 90 days of dialysis were included in this study. The dialysis modality on the 90^th^ day after the first service was considered as the initial modality. Patients were being censored for change of therapy or end of the study period, i.e. August 31, 2013. A total of 871 patients were included, of whom 641 initiated dialysis with HD and 230 with PD. For each patient, the baseline demographic characteristics (age, gender and ethnicity), co-morbid conditions at dialysis initiation (presence of diabetes mellitus [DM], hypertension, cardiovascular disease [CVD], and hyperlipidemia) and laboratory tests, such as left ventricular ejection fraction (LVEF), haemoglobin, serum albumin, phosphate, parathyroid hormone (PTH), alkaline phosphatase (ALP), calcium, urate, urea and estimated glomerular filtration rate (eGFR) were recorded. The postal codes of patients’ home addresses were retrieved from NUH to determine housing type (public residence vs. private residence), as a surrogate measure of socioeconomic status (SES). The information about death (died/alive, date of death and cause of death) was obtained from the National Registry of Disease Office under the approval of Ministry of Home Affairs (MHA), Singapore. Patients’ NRICs were used to match with the databases of MHA to retrieve the all-cause death information.

The Domain Specific Review Board, National Healthcare Group approved this study and waived the informed consent.

### Statistical analysis

The patient records data was de-identified and analyzed anonymously. Categorical variables were presented as frequency and percentage for the two groups (HD and PD) separately and compared using either chi-square test or Fisher’s exact test as appropriate. Continuous variables were presented as means ± standard deviation (SD) and compared using t-test. A two-sided p-value of less than 0.05 was considered statistically significant. Age was dichotomized into two groups (young [≤65-year-old]/old [>65-year-old]) in the analysis.

Survival analysis was performed using the flexible Royston-Parmar (RP) parametric model. The RP model is highly flexible alternative to the traditional Cox proportional hazards survival model when the assumption for the Cox model is violated [14]. In initial analysis, the Cox assumption was violated by diabetic status even when the time-varying effects were considered. Royston-Parmar model is able to parametrically model baseline survival functions and it has been shown to greatly improve the ability to accurately predict survival of some patient populations than the Cox model, so some researchers recommended using RP model in prognosticating patient survival [15, 16]. Univariate and multivariate RP survival analysis was performed using step-wise backward selection procedure with p<0.05 as the significance threshold.

The RP models were also estimated using a subsample of propensity score (PS) matched patients. The propensity score was the estimated probability of being treated initially with PD and was calculated using the multivariate logistic regression model. The matching technique was the nearest-neighbor matching within calipers without replacement with 1-to–1 matching (1 PD: 1 HD) and the caliper width was 0.2 of the SD of the logit of the propensity score [17]. This matching approach has been shown to result in the least biased treatment effect estimation among different PS-adjusted methods [18]. After matching, a subsample of 225 patient pairs was formed and then, we tested the balancing of propensity scores for each variable between HD and PD groups. The estimated mean bias in propensity score was 4.9% and 15.1% in the propensity score matched sample and in the raw sample (before matching), respectively.

The same RP model was further applied to subgroups defined by age and diabetes mellitus (young without DM, old without DM, young with DM, and old with DM) and by age and cardiovascular disease (young without CVD, old without CVD, young with CVD, and old with CVD).

The treatment effect on outcome was quantified using the hazard ratio (HR) estimate for PD patients, compared with HD patients, and its associated 95% confidence interval (CI). The values of HR >1 indicate a higher risk for death of PD than HD.

All analyses were performed using STATA (release 11.2; Stata Corp, College Station, TX, USA) statistical software.

## Results

Demographic and clinical characteristics are presented in Table 1. Patients initiating dialysis with PD (mean age: 64.3 years) were, on average, 6 years older than those initiating dialysis with HD (mean age: 58.2 years, p<0.001). There was higher proportion of females (57.4% versus 44.2%, p<0.01) and ethnic Chinese (66.1% versus 53.7%, p<0.01) in patients starting PD than those starting HD. At dialysis initiation, DM, hypertension, CVD and hyperlipidemia were more common among the PD patients. In terms of lab tests, patients who started dialysis with PD had higher hemoglobin levels, higher albumin concentrations, lower phosphate levels, and higher eGFR than those who started dialysis with HD.

**Table 1:** Patient characteristics at dialysis initiation.

	No. of patients		
	HD (n = 641)	PD (n = 230)	p-value
**Demographic**			
Age at diagnosis (y)	58.2±12.1	64.3±12.3	<0.001
≤65-year-old	454(70.8%)	115(50.0%)	<0.001
>65-year-old	187(29.2%)	115(50.0%)	
Gender (n)			<0.01
Male	358(55.8%)	98(42.6%)	
Female	283(44.2%)	132(57.4%)	
Ethnicity (n)			<0.01
Chinese	344(53.7%)	152(66.1%)	
Malay	211(32.9%)	58(25.2%)	
Indian & other	86(13.4%)	20(8.7%)	
Socioeconomic status (housing type) (n)			0.56
Lower (public residence)	594(92.7%)	210(91.3%)	
Higher (private residence)	47(7.3%)	20(8.7%)	
**Co-morbidity**			
Diabetes mellitus (%)	426(66.5%)	172(74.8%)	0.02
Hypertension (%)	587(91.6%)	219(95.2%)	0.08
Cardiovascular disease (%)	288(44.9%)	132(57.4%)	0.001
Hyperlipidemia (%)	145(22.6%)	58(25.2%)	0.47
**Laboratory tests**			
Left ventricular ejection fraction (LVEF)	55.5±13.6	53.3±15.5	0.04
Haemoglobin level (g/dL)	9.06±1.56	9.60±1.60	<0.001
Serum albumin level (g/L)	32.2±5.93	34.3±5.82	<0.001
Phosphate level (mmol/L)	2.00±0.76	1.85±0.82	<0.01
Parathyroid hormone (PTH) (pmol/L)	31.3±28.8	29.4±24.6	0.38
Alkaline phosphatase (ALP) (U/L)	96.7±65.4	91.0±49.7	0.23
Calcium level (mmol/L)	2.20±0.33	2.20±0.28	0.95
Urate level (μmol/L)	526.4±163.0	538.5±165.5	0.34
Urea level (mmol/L)	31.38±12.20	30.28±13.10	0.25
eGFR (ml/min/1.73m^2^)	7.25±4.24	8.63±5.63	<0.001

HD: hemodialysis, PD: peritoneal dialysis

**Fig 1:**
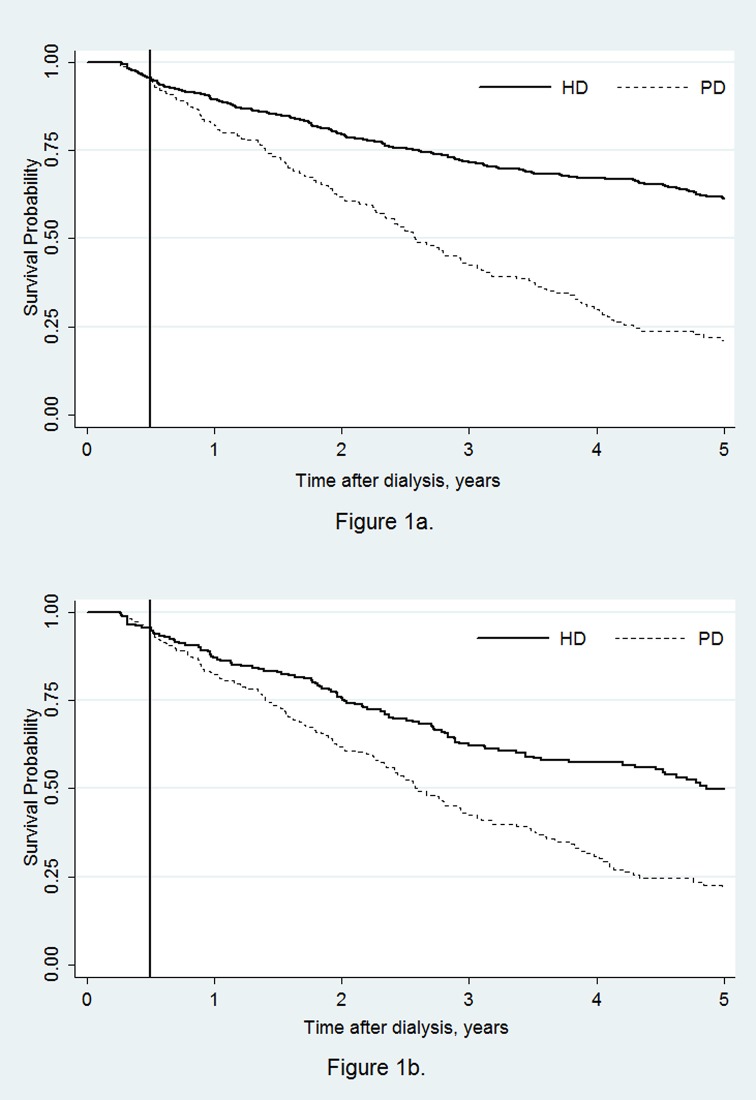
Kaplan-Meier survival curves by initial modality from day 90, using the entire cohort (Fig 1a, HD: 641, PD: 230) and the propensity score matched subsample (Fig 1b, HD: 225, PD: 225).

During the 5-year follow-up period, there were 225 deaths among the patients initiating HD (mortality rate: 7.02%) and 157 deaths among patients initiating PD (mortality rate: 13.7%). Fig 1a shows that the mortality rates for patients starting dialysis with the two modalities were similar in the first 6 months (p = 0.79, log-rank test) but started to differ after 6 months, with the mortality among those treated with PD initially being increasing at a higher rate.

**Fig 2:**
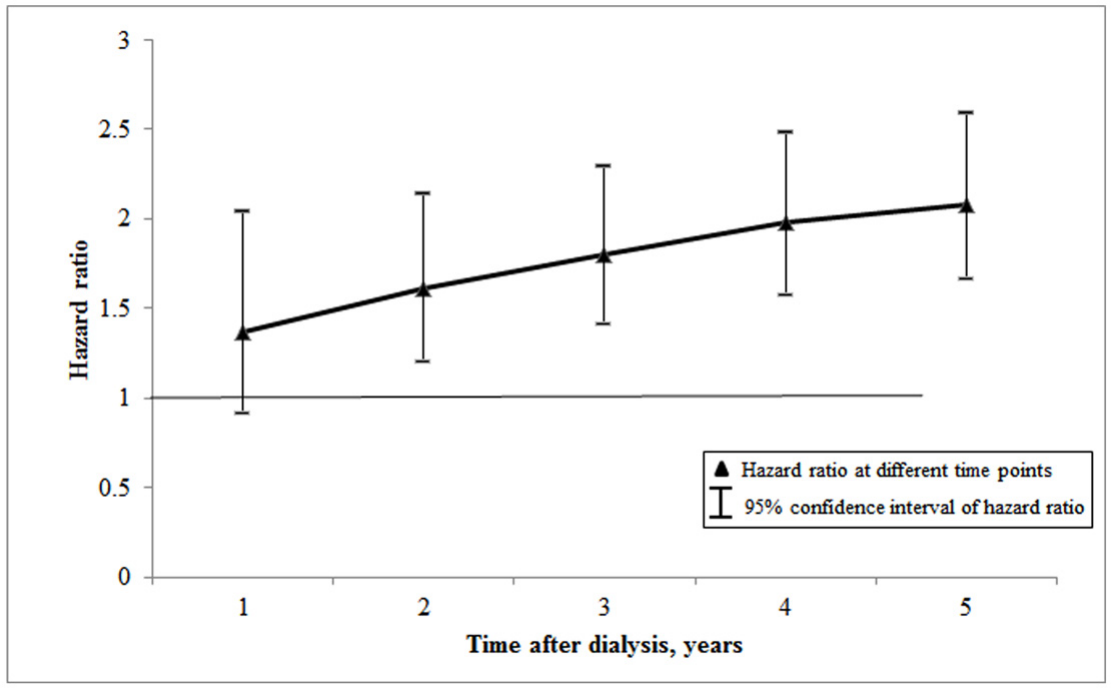
Hazard ratios for risk of death for patients initiating PD compared with those initiating HD and 95% confidence intervals using flexible RP model after adjusting for the effect of socio-demographic and clinical characteristics.

In multivariate flexible Royston-Parmar models, the risk of death was higher in patients starting PD than those starting HD (adjusted HR: 2.08; 95% CI: 1.67–2.59; p<0.001). Other significant predictors of higher risk of death were old age at diagnosis (>65-year-old) and presence of co-morbidity such as DM and CVD at the time of dialysis initiation (Table 2). Fig 2 shows that the adjusted HR of death of patients initiating PD compared to patients initiating HD was significantly higher than 1 during the follow-up period except for the first 12 months of treatment (HR: 1.37; 95% CI: 0.91–2.04; p = 0.13).

**Table 2:** Hazard ratios for risk of death for patients initiating PD compared with those initiating HD at 5-year follow-up.

Risk factor	HR	95% CI	p-value
PD modality	2.08	1.67–2.59	<0.001
Age group (>65 years)	1.85	1.50–2.27	<0.001
Diabetes mellitus (DM)	1.54	1.20–1.99	0.001
Cardiovascular disease (CVD)	2.06	1.65–2.56	<0.001

HR: hazard ratio, HD: hemodialysis, PD: peritoneal dialysis

In the subsample of 225 propensity score-matched patient pairs, the HR value was 1.73 (95% CI: 1.30–2.28, p<0.001), indicating that the patients initiating dialysis with PD had a higher risk of death than those initiating dialysis with HD. The Kaplan-Meier curves for this matched sample are presented in Fig 1b.

**Fig 3:**
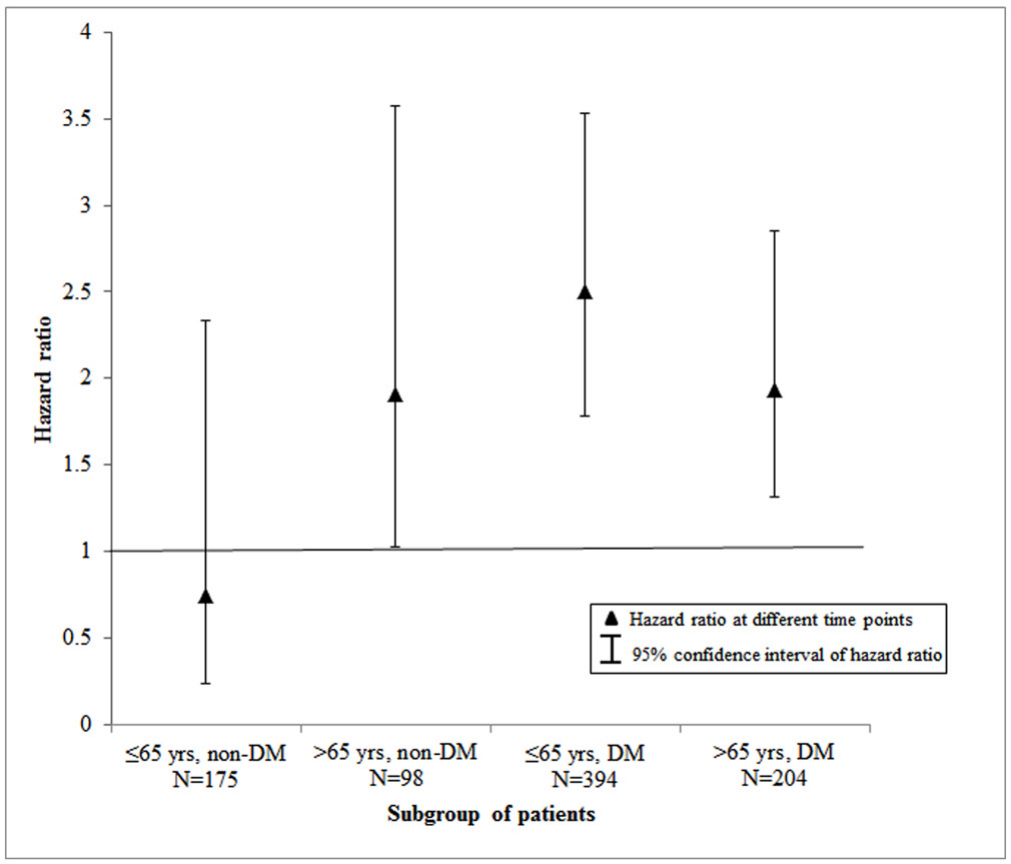
Hazard ratio for risk of death during 5-year follow-up for patients initiating PD compared with those initiating HD and 95% confidence intervals, stratified by age and the presence of DM.

**Fig 4:**
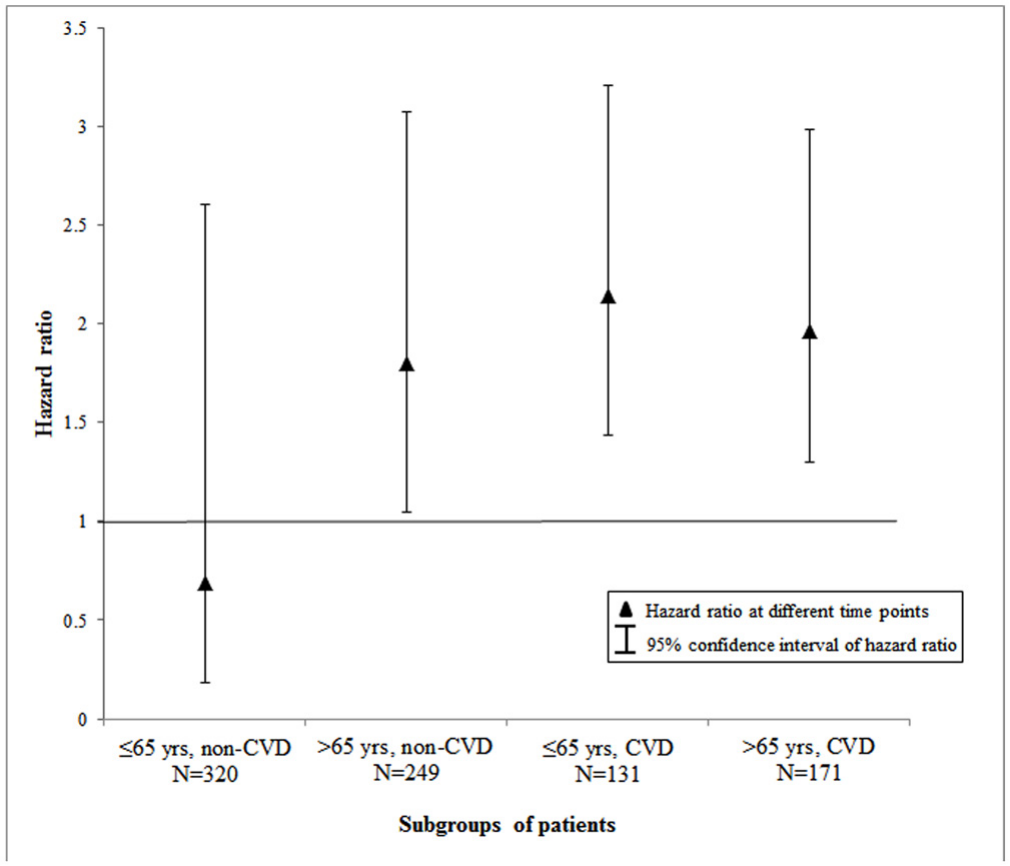
Hazard ratio for risk of death during 5-year follow-up for patients initiating PD compared with those initiating HD and 95% confidence intervals, stratified by age and the presence of CVD.

In subgroup analyses, the higher risk of death in patients starting dialysis with PD was observed in old patients without DM, young patients with DM, and old patients with DM during the 5 years of follow-up (Fig 3); however, among 175 young patients without DM (HD: 147, PD: 28), the risk of death did not differ between patients treated initially with PD and those treated initially with HD (adjusted HR: 0.75; 95% CI: 0.24–2.34; p = 0.62). Similar results were observed for subgroups defined by age and CVD (Fig 4). Patients initiating PD had significantly higher risk of death in all groups except for 320 young patients without CVD (HD: 266, PD: 54) (adjusted HR: 0.69; 95% CI: 0.18–2.61; p = 0.59).

## Discussion

In this study, we found that the survival outcomes may not differ between patients starting dialysis in the modality of PD and HD during the first year of treatment, but in the long term, the risk of death was significantly higher in patients initiating dialysis with PD. We also found that young (≤65-year-old) patients without diabetes mellitus or cardiovascular disease might benefit more from PD than HD.

Previous studies comparing the mortality of patients on PD and HD have shown varying results [6–9, 11–13, 19–21]. Several studies showed a higher risk of death for patients with PD after the first few years of treatment [6, 7, 9, 11], consistent with our findings, while some previous studies using either large-scale registry data or prospective cohort studies have revealed either better survival on PD in the first period on dialysis [8, 20] or comparable outcomes between the two treatment groups [12, 13, 19, 21].

The differing findings for the relative survival outcomes of the two dialysis modalities in the literature may be explained by several reasons. Firstly, ethnic difference is one possibility [22]. For example, diabetes mellitus is more common in Asian than Caucasian dialysis patients [23, 24]. The prevalence of diabetes mellitus was about 70% in this study and 50% in a Korean study which also found disadvantaged survival outcomes in PD patients [11]. In contrast, the prevalence of diabetes mellitus in Western dialysis patients ranged from 20% to 45% [7, 8, 10, 21, 25]. In addition, it was hypothesized that some Asian patient populations may be more likely to develop diabetes or worsened hyperglycemia during PD treatment because of the glucose-containing dialysis fluid [22].

Secondly, the variation in quality of PD may contribute. Factors such as peritoneal catheters and dialysis fluids used may affect the efficiency and quality of dialysis services [22]. Additionally, PD is monitored by the patient him/herself or a family member, and PD patients go to visit their health care providers infrequently. As a result, infections and complications may be less recognized and timely attended in patients undergoing PD than those undergoing HD in dialysis centers [7].

Thirdly, the differences may be due to selection bias. Nephrologists may tend to recommend PD to patients who have poor prognosis due to weak cardiac function [26], especially those with poor performance status, i.e. assisted activities of daily living or unable to ambulate, which would have precluded them from HD [27]. The better outcomes of HD may also be due to patients’ better economic status. Patients in better economic status may prefer HD because they are less concerned about the loss of productivity due to the treatment [5, 28].

It is not surprising that PD may perform similarly or better in young patients without diabetes mellitus or cardiovascular diseases than HD since consistent results have been reported in many previous studies [7, 11, 13, 29]. For example, the survival outcome of PD and HD was found to be comparable for non-diabetic patients under 55 years in Taiwan [13]. PD may confer a survival advantage to young and healthier patients due to better preservation of residual renal function compared to those undergoing HD [29], and therefore has been suggested to be largely used in the young healthier patients, particularly in countries where the PD utilization rate is low [25]. Moreover, PD has been shown to have advantages in patient satisfaction and quality of life [30, 31]. For example, a previous study of dialysis patients in Singapore [31] showed that patients undergoing PD perceived less burden of kidney disease and dialysis than those undergoing HD. Given the relatively low costs of PD and comparable outcomes, promoting PD in these subgroups is likely to lead to cost-effective care. As the survival advantage of PD in the young healthier patients in our study is not certain, further investigation is warranted.

The limitations of the study should not be overlooked. First, because of the lack of randomized controlled clinical trials, such observational study could only provide information on the effectiveness of dialysis modalities instead of causality between dialysis modality and mortality. Second, despite the efforts made to adjust for socio-demographic and clinical characteristics, there may be unmeasured differences between HD and PD patients, which might lead to excess baseline risk for PD patients. Previous studies have shown that in countries where older and sicker patients are preferentially considered for PD [6], patients treated with PD have a higher risk of death than those treated with HD after adjusting for covariates; while in countries demographic and comorbidity data was comparable in both groups of patients, the disadvantage of PD was not observed. Third, the vascular access type of HD patients was not available. HD vascular access type was reported to be strongly associated with the prognosis of patients [32], and thus comparisons of HD and PD should include such information whenever possible. Last, the data used here was from a single-center study; it may not reflect the dialysis outcomes in other study sites. In view of these drawbacks, caution should be exercised in generalizing the results until further studies could confirm the findings.

In conclusion, there was a survival advantage of patients who initiated HD during 5 years of follow-up. Survival outcomes may not differ between the two dialysis modalities in young and healthier patients and further work is needed to evaluate the possible survival benefit of PD as the first treatment in these patients. These findings are potentially confounded by selection bias as patients were not randomized to the two dialysis modalities in this cohort study.
